# Validation of an Automated System for the Extraction of a Wide Dataset for Clinical Studies Aimed at Improving the Early Diagnosis of Candidemia

**DOI:** 10.3390/diagnostics13050961

**Published:** 2023-03-03

**Authors:** Daniele Roberto Giacobbe, Sara Mora, Alessio Signori, Chiara Russo, Giorgia Brucci, Cristina Campi, Sabrina Guastavino, Cristina Marelli, Alessandro Limongelli, Antonio Vena, Malgorzata Mikulska, Anna Marchese, Antonio Di Biagio, Mauro Giacomini, Matteo Bassetti

**Affiliations:** 1 Department of Health Sciences (DISSAL), University of Genoa, 16132 Genoa, Italy; 2 Clinica Malattie Infettive, IRCCS Ospedale Policlinico San Martino, 16132 Genoa, Italy; 3 Department of Informatics, Bioengineering, Robotics and System Engineering (DIBRIS), University of Genoa, 16145 Genoa, Italy; 4 Section of Biostatistics, Department of Health Sciences (DISSAL), University of Genoa, 16132 Genoa, Italy; 5 Department of Mathematics (DIMA), University of Genoa, 16146 Genoa, Italy; 6 IRCCS Ospedale Policlinico San Martino, 16132 Genoa, Italy; 7 Department of Surgical Sciences and Integrated Diagnostics (DISC), University of Genoa, 16132 Genoa, Italy; 8 Microbiology Unit, IRCCS Ospedale Policlinico San Martino, 16132 Genoa, Italy

**Keywords:** *Candida*, early diagnosis, machine learning, relational database, bloodstream infections

## Abstract

There is increasing interest in assessing whether machine learning (ML) techniques could further improve the early diagnosis of candidemia among patients with a consistent clinical picture. The objective of the present study is to validate the accuracy of a system for the automated extraction from a hospital laboratory software of a large number of features from candidemia and/or bacteremia episodes as the first phase of the AUTO-CAND project. The manual validation was performed on a representative and randomly extracted subset of episodes of candidemia and/or bacteremia. The manual validation of the random extraction of 381 episodes of candidemia and/or bacteremia, with automated organization in structured features of laboratory and microbiological data resulted in ≥99% correct extractions (with confidence interval < ±1%) for all variables. The final automatically extracted dataset consisted of 1338 episodes of candidemia (8%), 14,112 episodes of bacteremia (90%), and 302 episodes of mixed candidemia/bacteremia (2%). The final dataset will serve to assess the performance of different ML models for the early diagnosis of candidemia in the second phase of the AUTO-CAND project.

## 1. Introduction

Bloodstream infections (BSIs) due to *Candida* spp. (candidemia) are among the most frequent BSIs encountered in hospitalized patients and remain associated with high mortality, especially in critically ill patients and when presenting as septic shock [1,2,3,4,5].

The clinical presentation of candidemia is not associated with highly specific signs and symptoms and is frequently indistinguishable from that of bacteremia, which is overall more frequent [6,7]. While waiting for blood culture results, which could take up to 48–72 h, two core aspects in the bedside management of patients with suspected candidemia are the following: (i) to guarantee an efficacious early antifungal treatment for true cases (i.e., patients who are later confirmed to truly have candidemia); (ii) to avoid the use of antifungals in patients with bacteremia only, in whom, from an antifungal stewardship standpoint, it would be more appropriate to administer only empiric antibacterials (and to rapidly discontinue empirical antifungals if already initiated) [8,9,10]. Since the clinical presentations of candidemia and bacteremia are very similar, clinicians usually rely on clinical scores and serum laboratory markers for rapidly identifying those patients who require or do not require early antifungal treatment [11,12,13,14,15,16,17,18,19,20,21,22,23,24,25,26,27].

Clinical scores and biomarkers-based approaches are very useful, but are still far from perfection. This is one of the possible reasons why there is increasing interest in exploring whether machine learning (ML) models could further improve the early recognition of candidemia in patients with a consistent clinical picture [28,29]. Since some ML models require large datasets in terms of both training examples and number of features (to achieve sufficient accuracy), an important pre-requisite for reliably exploring the role of these techniques is the ability to build very large datasets [30,31,32]. This may represent an extremely time-consuming task if performed manually and could be impossible to fulfill in various real-life situations. This crucial limitation could be overcome by exploiting efficient automated systems for the accurate extraction and organization of features from hospitals’ laboratory data and electronic health records.

In the present study, we aimed to validate the accuracy of a system for the automated extraction from a hospital laboratory information system (LIS) of a large number of features from candidemia and/or bacteremia episodes. The extracted dataset will serve to assess the performance of different ML models for the early diagnosis of candidemia within the AUTO-CAND project. The primary objective of the AUTO-CAND project is to assess the diagnostic performance of different supervised ML methods for the differential diagnosis between candidemia and bacteremia, exploiting a large database of automatically extracted candidemia and bacteremia episodes.

## 2. Methods

### 2.1. Setting and Objective

**Figure 1:**
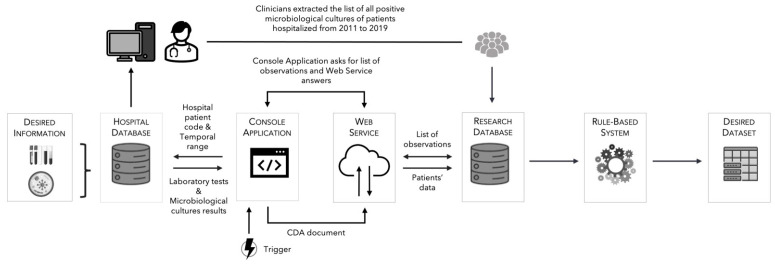
Architecture of the automated extraction system. Legend: Graphical representation of the architecture and sequence of events that lead to the creation of the desired dataset. The events chain that leads the desired information from the hospital LIS towards the research database is regulated by a trigger. The trigger was executed once each night until the data collection phase was completed. Specifically, the console application obtained by the web services the list of episodes, each of them linked to a specific patient and a time span. For each episode, a query is executed in the ad-hoc view of the hospital database in order to read laboratory tests and microbiological culture results. These data are then transferred and stored into the research database. Last, a rule-based system extracts the desired features from the research database and organizes them into the final dataset.

The present retrospective study was conducted at IRCCS Ospedale Policlinico San Martino, a 1200-bed teaching hospital in Italy, and represents the first phase of the AUTO-CAND project. An automated extraction system was developed for extracting and organizing data pertaining to single episodes of candidemia and/or bacteremia (thereby also including mixed episodes of candidemia plus bacteremia) occurred between 1 January 2011 and 31 December 2019. The complete architecture of the developed system is summarized graphically in Figure 1, and it can be divided into two main chunks. The first one is devoted to the automated extraction and transfer of data from hospital LIS to the project research database. Its preliminary architecture has been presented at the European Federation for Medical Informatics (EFMI) congress in 2021 [33]. In brief, a Windows Console Application (client) reads and organizes laboratory data from an ad-hoc view on the hospital LIS into a document compliant to the Clinical Document Architecture Release 2 (CDA R2) standard. The database view exposes already pseudonymized data, thus only the hospital code of the patient is present and is used to identify the results of laboratory procedures belonging to him/her. The information contained in other database fields (e.g., date, exam code, value, etc.) was used to build a complete picture of the extracted information about the laboratory procedure. First, the local identifier of the laboratory procedure and the corresponding translation were put into the international coding system, which Italian regulation requires to be the logical observation identifiers’ names and codes (LOINC). This information is used to uniquely identify each exam and keep it tracked over possible modifications that could happen locally over the years. Then, each laboratory test result is stored together with the unit of measure and the reference range. The CDA R2 is then received by a main Windows Communication Foundation service which extracts and stores the features in the target database after validation of the document structure [33]. The system exploits some modules previously developed for the Liguria Infectious Diseases Network, described in [34,35], whose architecture was developed in line with service-oriented approaches [36]. The second element of the architecture is a rule-based system. Its main aim is to read data from the research database and perform the necessary re-elaborations in order to extract and organize the desired list of features identified by expert medical staff as important for the scope.

The objective of the present study was to manually validate the extraction accuracy of the automated system on a representative and randomly extracted subset of candidemia and/or bacteremia episodes. Manual validation was deemed necessary to guarantee high accuracy of the subsequently performed automated extraction of the final dataset of all candidemia and/or bacteremia episodes that occurred during the study period. The final automatically extracted dataset will serve in the second phase of the AUTO-CAND project for assessing the performance of different ML models for the early recognition of candidemia. The project was approved by the local ethics committee (Liguria Region Ethics Committee, approval number 71/2020). Informed consent collection was waived due to the retrospective nature of the analyses.

### 2.2. Definitions

The developed automated extraction system is able to: (i) recognize the origin of each episode of candidemia and/or bacteremia; (ii) recognize different episodes occurring in the same patient; (iii) recognize mixed episodes of candidemia and bacteremia; (iv) differentiate bacteremia episodes by coagulase-negative staphylococci or other common skin colonizers from contamination. The origin of candidemia and bacteremia episodes was defined as the day, hour, and minute the first positive blood culture was collected and sent to the hospital laboratory. This information is routinely and automatically registered on the hospital LIS for each collected blood culture. An episode caused by the same *Candida* species or the same bacterial species responsible for a previous episode was considered as independent (i.e., as a different, novel episode) if it was developing at least 30 days after the collection of the last positive blood culture from the previous episode. A mixed candidemia/bacteremia episode was defined if the origins of candidemia and bacteremia in the same patient occurred less than 48 h apart [37]. An episode of bacteremia caused by coagulase-negative staphylococci or other common skin colonizers was defined as a true episode of bacteremia only if two positive blood cultures for the same organism were collected from two different sites/sets (i.e., from different body sites or from the same site at different times) less than 48 h apart [38].

### 2.3. Data Collected for the Study

The developed system is able to automatically extract and organize the results of laboratory blood tests in the target dataset performed at the origin and in the 7 days before the origin of each candidemia and/or bacteremia episode, as follows: (i) extracted laboratory results at day 0 for each test are the closest to the origin of the episode within a time window of −12 h to +2 h with respect to the origin; (ii) extracted laboratory results at day −1 for each test are the closest to the origin of the episode minus 24 h within a time window of −12 h to +12 h with respect to the origin minus 24 h; (iii) the same criterion for day −1 with respect to the previous day is employed for the extraction of laboratory results at day −2, −3, −4, −5, −6, and −7; (iv) if a specific laboratory test was not performed within the time window specified for a specific day, the value is considered as missing. The results of the following blood tests are automatically extracted from the laboratory software: white cells count; red cells count; platelet count; neutrophil cells count; lymphocyte cells count; basophil cells count; eosinophil cell counts; monocyte cells count; hemoglobin; hematocrit; creatinine; urea; uric acid; lactate; lactate dehydrogenase; alkaline phosphatase; gamma-glutamyl transferase; alanine aminotransferase; aspartate aminotransferase; total bilirubin; direct bilirubin; indirect bilirubin; activated partial thromboplastin time; prothrombin time; international normalized ratio; glucose; glycated hemoglobin; total proteins; albumin; triglycerides; C-reactive protein; procalcitonin; beta-D-glucan.

With regard to microbiological data, the developed system is able to automatically extract and organize in the following variables the results of cultures other than blood performed within 30 days before the origin of each candidemia and/or bacteremia episode: (i) respiratory colonization by *Candida* spp. and/or bacteria (genus and species for both *Candida* and bacteria); (ii) urinary colonization by *Candida* spp. and/or bacteria (genus and species for both *Candida* and bacteria); (iii) gastrointestinal colonization by *Candida* spp. and/or bacteria (genus and species for both *Candida* and bacteria); (iv) presence of *Candida* colonization (yes vs. no); (v) presence of multifocal *Candida* colonization; (vi) number of colonized sites. The system is also able to recognize whether a specific site of colonization was explored or not (i.e., when no cultures for that specific site were performed within the 30 days before the origin of the episode) in order to appropriately adjust future analyses.

### 2.4. Manual Validation Procedure

A total of 381 de-identified episodes of candidemia and/or bacteremia were randomly extracted by the automated system. For each automatically extracted variable (e.g., “was this patient with candidemia and/or bacteremia already colonized by *Candida* spp.?” yes/no), the medical doctors involved in the project (who had access to the alphanumeric key for patient identification from pseudonymized data) compared the extracted data with the original information on the laboratory software in order to perform manual validation (i.e., correct extraction yes/no) of each extracted value. The choice of 381 random episodes extraction was based on the desired uncertainty margin (95% confidence interval [CI] for proportions) of the validated accuracy. In more detail, assuming normal distribution of the estimated population parameter of a given variable (e.g., previous *Candida* colonization yes/no), the uncertainty margin for a ≥99% accuracy (i.e., ≥99% correctly extracted values of that variable) from 381 episodes would have been <±1%. We required extraction to be highly accurate for all variables as a necessary prerequisite for the subsequent automated extraction of all candidemia and/or bacteremia episodes in the final dataset. In case of accuracy <99% for any given variable, manual revision was stopped, and the extraction code was revised by the bioengineers involved in the project after discussion with clinicians on the possible nature of the extraction error/s, with a subsequent novel random extraction until the achievement of a proportion of correct automated extractions ≥ 99%.

## 3. Results

**Figure 2:**
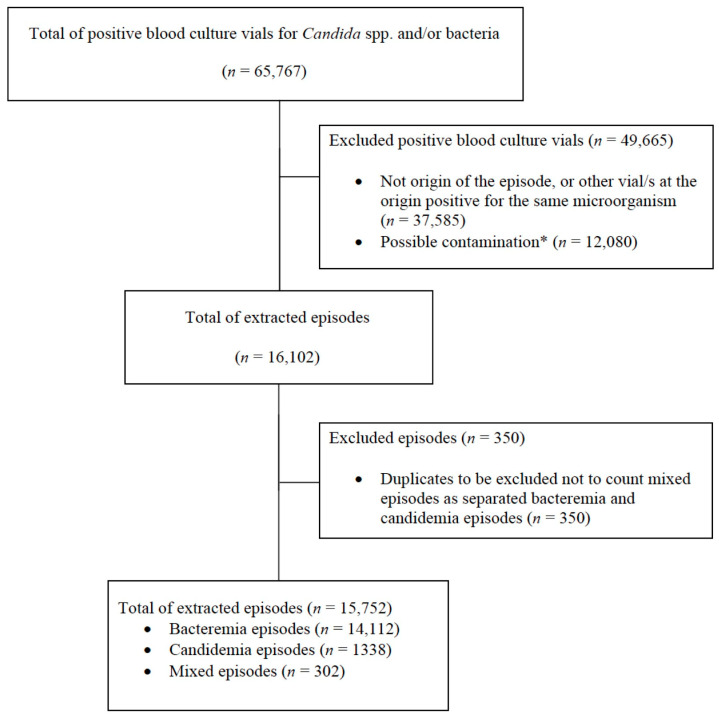
Flow-chart of the automated extraction process of candidemia and/or bacteremia episodes. Legend: * For the purposes of the AUTO-CAND project, we did not differentiate between the following: (i) contamination due to only one positive blood culture for common skin colonizers among multiple blood cultures performed; (ii) possible contamination when only one vial was sent to the laboratory and resulted positive for common skin colonizers. Indeed, the latter cases could represent true bacteremia episodes, but given the lack of certainty they also have to be excluded from the analyses of the second phase of the AUTO-CAND project. Should it be of interest for other future analyses/studies, the automated extraction system can be easily updated to also differentiate between (i) and (ii).

After three preliminary test extractions of 20 episodes each that allowed us to identify and correct some typo errors in the extraction code, the manual validation of the first complete random extraction of 381 episodes of candidemia and/or bacteremia resulted in ≥99% correct extractions for all the considered variables. The proportions of correct extractions for all variables as verified by manual validation are detailed in Supplementary Table S1. As reported in the table, there was a single case of incorrect extraction for only one variable (a urine culture was performed within 30 days before the origin of the given episode that yielded no organism growth, but the system did not extract this information and categorized colonization of the urinary tract as unexplored). Nonetheless, the rarity of this technical error allowed us to declare achievement of an acceptable accuracy of automated extraction. We then performed the final extraction of all candidemia and/or bacteremia episodes that occurred during the study period. As shown in Figure 2, the final automatically extracted dataset consisted of 1338 episodes of candidemia (8%), 14,112 episodes of bacteremia (90%), and 302 episodes of mixed candidemia/bacteremia (2%).

## 4. Discussion

The present study represented the first phase of the AUTO-CAND project, which aimed to assess the performance of different ML models (e.g., logistic regression, least absolute shrinkage and selection operator (LASSO) regression, support vector machines (SVM), random forest, and neural networks) for the early recognition of candidemia. The use of an automated data extraction system allowed for the creation of a large dataset, an extremely time-consuming task that would have been difficult or even impossible to achieve manually. However, it was essential to evaluate the quality of the dataset by comparing the data collected automatically with those that would have been collected manually [39,40]. This study reports the manual validation process that we performed to assess the performance of our extraction system. Moreover, despite the detection of a single technical error during manual validation, it is worth noting that the expected rate of errors during automated extraction is far lower than the one expected with manual imputation of data [41,42]. Certainly, the extraction and organization of data performed automatically by our system is currently limited to laboratory and microbiological variables, while it is well-known from the literature that other clinical variables (e.g., comorbidities, use of invasive devices, previous use of broad-spectrum antibiotics) could contribute to the risk of candidemia (and thus also influencing the probability of candidemia when combined in clinical scores used as early diagnostic tools in patients with consistent signs and symptoms) [7,20,23]. In this regard, we think two points should be discussed. The first is that it cannot be excluded a priori that a ML model trained on a large number of laboratory and microbiological variables could be already sufficiently accurate in predicting candidemia; thus, in our opinion, this possibility is worth testing [31]. The second point is that our group is concomitantly working on the development of a natural language processing (NLP)-based pipeline for the extraction of clinical variables from the text of laboratory notes and electronic health records [43], that, in the future, could expand our ability to automatically extract relevant features beyond laboratory and microbiological variables.

From a clinical perspective, a limitation of our extraction system in its current form is that it cannot define a candidemia and/or bacteremia episode based on signs and symptoms of infection, but only based on the results of blood cultures. Furthermore, it should be acknowledged that we arbitrarily decided how to categorize the day of laboratory results (e.g., 0, −1, −2, −7) and the timeframes for variables collection (i.e., seven days before the origin of the episode for blood tests and thirty days before the origin of the episode for microbiological cultures). However, it is worth noting that, to the best of our knowledge, there are currently no rules or guidelines on how to define such categorizations and timeframes. Therefore, an arbitrary decision was eventually unavoidable. We opted for a categorization based on the distance from the origin in terms of day, since this could remain intuitive for investigators dealing with the extracted dataset, either for checking data or for analysis purposes. In any case, the results in terms of performance of ML models in the second phase of the AUTO-CAND project will also help us to confirm the validity of our arbitrary rules and to assess whether or not the dataset architecture should be revised. Finally, our project will compare patients with candidemia vs. patients with bacteremia, and not patients with candidemia vs. patients with negative blood cultures and a clinical picture consistent with candidemia, which are also encountered in daily practice. However, this is not a limitation, considering that such patients may have an undetected fungal or bacterial infection. Consequently, future studies will need to evaluate whether ML models trained on a dataset of patients with positive blood cultures, including ours, could guide clinicians in properly selecting early antifungal/antibacterial therapy in patients with negative blood cultures.

In conclusion, we validated the accuracy of an automated extraction system of laboratory and microbiological variables from patients with candidemia and/or bacteremia. The extracted dataset will serve for assessing the performance of different ML models for the early recognition of candidemia. Future improvements of the system through the implementation of NLP-based algorithms could expand the number and types of extracted features, as well as its applicability to other fields of medical research.

## Data Availability

The data presented in this study will be available from the corresponding author for post-hoc analyses after conclusion of the entire project, on reasonable request and provided all regulatory and privacy requirements are fulfilled.

## Supplementary Materials

The following supporting information can be downloaded at: https://www.mdpi.com/article/10.3390/diagnostics13050961/s1, Table S1: Manual validation of extraction accuracy.
