# Supplementary material for: Validation of an Automated System for the Extraction of a Wide Dataset for Clinical Studies Aimed at Improving the Early Diagnosis of Candidemia
Source: Diagnostics (Basel). 2023 Mar 3;13(5):961. doi: 10.3390/diagnostics13050961 (PMC10001256; doi:10.3390/diagnostics13050961)
Supplement: Supplementary file 1 [file diagnostics-13-00961-s001.zip › diagnostics-2227954-supplementary.pdf]

Supplementary table S1. Manual validation of extraction accuracy*

Feature	Number of correct extractions	Total extractions	Percentage of correct extractions
Candidemia episode (yes/no)	381	381	100.00%
Bacteremia episode (yes/no)	381	381	100.00%
Correct origin of the episode	381	381	100.00%
Contamination (yes/no)**	381	381	100.00%
Candida colonization in the previous 30 days (yes/no)	381	381	100.00%
Bacterial colonization in the previous 30 days (yes/no)	381	381	100.00%
Multifocal *Candida* colonization (yes/no)	381	381	100.00%
Number of explored body sites for colonization	380	381	99.74%
Respiratory colonization by *Candida* spp. (yes/no)	381	381	100.00%
Respiratory colonization by bacteria (yes/no)	381	381	100.00%
Genus and species of respiratory isolates	381	381	100.00%
Urinary colonization by *Candida* spp. (yes/no)	381	381	100.00%
Urinary colonization by bacteria (yes/no)	381	381	100.00%
Genus and species of urinary isolates	381	381	100.00%
Gastrointestinal colonization by *Candida* spp. ~~only~~ (yes/no)	381	381	100.00%
Gastrointestinal colonization by bacteria (yes/no)	381	381	100.00%
Genus and species of gastrointestinal isolates	381	381	100.00%
Basophil cells count (day 0)	381	381	100.00%
Eosinophil cells count (day 0)	381	381	100.00%
Lymphocyte cells count (day 0)	381	381	100.00%
Monocyte cells count (day 0)	381	381	100.00%
Neutrophil cells count (day 0)	381	381	100.00%
Hematocrit (day 0)	381	381	100.00%
Hemoglobin (day 0)	381	381	100.00%
White cells count (day 0)	381	381	100.00%
Red cells count (day 0)	381	381	100.00%
Platelet count (day 0)	381	381	100.00%
Activated partial thromboplastin time (day 0)	381	381	100.00%
International normalized ratio (day 0)	381	381	100.00%
Prothrombin time (day 0)	381	381	100.00%
Uric acid (day 0)	381	381	100.00%
Alkaline phosphatase (day 0)	381	381	100.00%
Alanine aminotransferase (day 0)	381	381	100.00%
Aspartate aminotransferase (day 0)	381	381	100.00%
Direct bilirubin (day 0)	381	381	100.00%
Total bilirubin (day 0)	381	381	100.00%
Creatinine (day 0)	381	381	100.00%
Gamma-glutamyl transferase (day 0)	381	381	100.00%
Lactate dehydrogenase (day 0)	381	381	100.00%
Urea (day 0)	381	381	100.00%

Glycated hemoglobin (day 0)	381	381	100.00%
Glucose (day 0)	381	381	100.00%
Albumin (day 0)	381	381	100.00%
Beta-D-glucan (day 0)	381	381	100.00%
C-reactive protein (day 0)	381	381	100.00%
Procalcitonin (day 0)	381	381	100.00%
Total proteins (day 0)	381	381	100.00%
Lactate from arterial blood (day 0)	381	381	100.00%
Lactate from venous blood (day 0)	381	381	100.00%
Triglycerides (day 0)	381	381	100.00%
Basophil cells count (day -1)	381	381	100.00%
Eosinophil cells count (day -1)	381	381	100.00%
Lymphocyte cells count (day -1)	381	381	100.00%
Monocyte cells count (day -1)	381	381	100.00%
Neutrophil cells count (day -1)	381	381	100.00%
Hematocrit (day -1)	381	381	100.00%
Hemoglobin (day -1)	381	381	100.00%
White cells count (day -1)	381	381	100.00%
Red cells count (day -1)	381	381	100.00%
Platelet count (day -1)	381	381	100.00%
Activated partial thromboplastin time (day -1)	381	381	100.00%
International normalized ratio (day -1)	381	381	100.00%
Prothrombin time (day -1)	381	381	100.00%
Uric acid (day -1)	381	381	100.00%
Alkaline phosphatase (day -1)	381	381	100.00%
Alanine aminotransferase (day -1)	381	381	100.00%
Aspartate aminotransferase (day -1)	381	381	100.00%
Direct bilirubin (day -1)	381	381	100.00%
Total bilirubin (day -1)	381	381	100.00%
Creatinine (day -1)	381	381	100.00%
Gamma-glutamyl transferase (day -1)	381	381	100.00%
Lactate dehydrogenase (day -1)	381	381	100.00%
Urea (day -1)	381	381	100.00%
Glycated hemoglobin (day -1)	381	381	100.00%
Glucose (day -1)	381	381	100.00%
Albumin (day -1)	381	381	100.00%
Beta-D-glucan (day -1)	381	381	100.00%
C-reactive protein (day -1)	381	381	100.00%
Procalcitonin (day -1)	381	381	100.00%
Total proteins (day -1)	381	381	100.00%
Lactate from arterial blood (day -1)	381	381	100.00%
Lactate from venous blood (day -1)	381	381	100.00%
Triglycerides (day -1)	381	381	100.00%
Basophil cells count (day -2)	381	381	100.00%
Eosinophil cells count (day -2)	381	381	100.00%
Lymphocyte cells count (day -2)	381	381	100.00%

Monocyte cells count (day -2)	381	381	100.00%
Neutrophil cells count (day -2)	381	381	100.00%
Hematocrit (day -2)	381	381	100.00%
Hemoglobin (day -2)	381	381	100.00%
White cells count (day -2)	381	381	100.00%
Red cells count (day -2)	381	381	100.00%
Platelet count (day -2)	381	381	100.00%
Activated partial thromboplastin time (day -2)	381	381	100.00%
International normalized ratio (day -2)	381	381	100.00%
Prothrombin time (day -2)	381	381	100.00%
Uric acid (day -2)	381	381	100.00%
Alkaline phosphatase (day -2)	381	381	100.00%
Alanine aminotransferase (day -2)	381	381	100.00%
Aspartate aminotransferase (day -2)	381	381	100.00%
Direct bilirubin (day -2)	381	381	100.00%
Total bilirubin (day -2)	381	381	100.00%
Creatinine (day -2)	381	381	100.00%
Gamma-glutamyl transferase (day -2)	381	381	100.00%
Lactate dehydrogenase (day -2)	381	381	100.00%
Urea (day -2)	381	381	100.00%
Glycated hemoglobin (day -2)	381	381	100.00%
Glucose (day -2)	381	381	100.00%
Albumin (day -2)	381	381	100.00%
Beta-D-glucan (day -2)	381	381	100.00%
C-reactive protein (day -2)	381	381	100.00%
Procalcitonin (day -2)	381	381	100.00%
Total proteins (day -2)	381	381	100.00%
Lactate from arterial blood (day -2)	381	381	100.00%
Lactate from venous blood (day -2)	381	381	100.00%
Triglycerides (day -2)	381	381	100.00%
Basophil cells count (day -3)	381	381	100.00%
Eosinophil cells count (day -3)	381	381	100.00%
Lymphocyte cells count (day -3)	381	381	100.00%
Monocyte cells count (day -3)	381	381	100.00%
Neutrophil cells count (day -3)	381	381	100.00%
Hematocrit (day -3)	381	381	100.00%
Hemoglobin (day -3)	381	381	100.00%
White cell count (day -3)	381	381	100.00%
Red cell count (day -3)	381	381	100.00%
Platelet count (day -3)	381	381	100.00%
Activated partial thromboplastin time (day -3)	381	381	100.00%
International normalized ratio (day -3)	381	381	100.00%
Prothrombin time (day -3)	381	381	100.00%
Uric acid (day -3)	381	381	100.00%
Alkaline phosphatase (day -3)	381	381	100.00%
Alanine aminotransferase (day -3)	381	381	100.00%

Aspartate aminotransferase (day -3)	381	381	100.00%
Direct bilirubin (day -3)	381	381	100.00%
Total bilirubin (day -3)	381	381	100.00%
Creatinine (day -3)	381	381	100.00%
Gamma-glutamyl transferase (day -3)	381	381	100.00%
Lactate dehydrogenase (day -3)	381	381	100.00%
Urea (day -3)	381	381	100.00%
Glycated hemoglobin (day -3)	381	381	100.00%
Glucose (day -3)	381	381	100.00%
Albumin (day -3)	381	381	100.00%
Beta-D-glucan (day -3)	381	381	100.00%
C-reactive protein (day -3)	381	381	100.00%
Procalcitonin (day -3)	381	381	100.00%
Total proteins (day -3)	381	381	100.00%
Lactate from arterial blood (day -3)	381	381	100.00%
Lactate from venous blood (day -3)	381	381	100.00%
Triglycerides (day -3)	381	381	100.00%
Basophil cells count (day -4)	381	381	100.00%
Eosinophil cells count (day -4)	381	381	100.00%
Lymphocyte cells count (day -4)	381	381	100.00%
Monocyte cells count (day -4)	381	381	100.00%
Neutrophil cells count (day -4)	381	381	100.00%
Hematocrit (day -4)	381	381	100.00%
Hemoglobin (day -4)	381	381	100.00%
White cells count (day -4)	381	381	100.00%
Red cells count (day -4)	381	381	100.00%
Platelet count (day -4)	381	381	100.00%
Activated partial thromboplastin time (day -4)	381	381	100.00%
International normalized ratio (day -4)	381	381	100.00%
Prothrombin time (day -4)	381	381	100.00%
Uric acid (day -4)	381	381	100.00%
Alkaline phosphatase (day -4)	381	381	100.00%
Alanine aminotransferase (day -4)	381	381	100.00%
Aspartate aminotransferase (day -4)	381	381	100.00%
Direct bilirubin (day -4)	381	381	100.00%
Total bilirubin (day -4)	381	381	100.00%
Creatinine (day -4)	381	381	100.00%
Gamma-glutamyl transferase (day -4)	381	381	100.00%
Lactate dehydrogenase (day -4)	381	381	100.00%
Urea (day -4)	381	381	100.00%
Glycated hemoglobin (day -4)	381	381	100.00%
Glucose (day -4)	381	381	100.00%
Albumin (day -4)	381	381	100.00%
Beta-D-glucan (day -4)	381	381	100.00%
C-reactive protein (day -4)	381	381	100.00%
Procalcitonin (day -4)	381	381	100.00%

Total proteins (day -4)	381	381	100.00%
Lactate from arterial blood (day -4)	381	381	100.00%
Lactate from venous blood (day -4)	381	381	100.00%
Triglycerides (day -4)	381	381	100.00%
Basophil cells count (day -5)	381	381	100.00%
Eosinophil cells count (day -5)	381	381	100.00%
Lymphocyte cells count (day -5)	381	381	100.00%
Monocyte cells count (day -5)	381	381	100.00%
Neutrophil cells count (day -5)	381	381	100.00%
Hematocrit (day -5)	381	381	100.00%
Hemoglobin (day -5)	381	381	100.00%
White cells count (day -5)	381	381	100.00%
Red cells count (day -5)	381	381	100.00%
Platelet count (day -5)	381	381	100.00%
Activated partial thromboplastin time (day -5)	381	381	100.00%
International normalized ratio (day -5)	381	381	100.00%
Prothrombin time (day -5)	381	381	100.00%
Uric acid (day -5)	381	381	100.00%
Alkaline phosphatase (day -5)	381	381	100.00%
Alanine aminotransferase (day -5)	381	381	100.00%
Aspartate aminotransferase (day -5)	381	381	100.00%
Direct bilirubin (day -5)	381	381	100.00%
Total bilirubin (day -5)	381	381	100.00%
Creatinine (day -5)	381	381	100.00%
Gamma-glutamyl transferase (day -5)	381	381	100.00%
Lactate dehydrogenase (day -5)	381	381	100.00%
Urea (day -5)	381	381	100.00%
Glycated hemoglobin (day -5)	381	381	100.00%
Glucose (day -5)	381	381	100.00%
Albumin (day -5)	381	381	100.00%
Beta-D-glucan (day -5)	381	381	100.00%
C-reactive protein (day -5)	381	381	100.00%
Procalcitonin (day -5)	381	381	100.00%
Total proteins (day -5)	381	381	100.00%
Lactate from arterial blood (day -5)	381	381	100.00%
Lactate from venous blood (day -5)	381	381	100.00%
Triglycerides (day -5)	381	381	100.00%
Basophil cells count (day -6)	381	381	100.00%
Eosinophil cells count (day -6)	381	381	100.00%
Lymphocyte cells count (day -6)	381	381	100.00%
Monocyte cells count (day -6)	381	381	100.00%
Neutrophil cells count (day -6)	381	381	100.00%
Hematocrit (day -6)	381	381	100.00%
Hemoglobin (day -6)	381	381	100.00%
White cells count (day -6)	381	381	100.00%
Red cells count (day -6)	381	381	100.00%

Platelet count (day -6)	381	381	100.00%
Activated partial thromboplastin time (day -6)	381	381	100.00%
International normalized ratio (day -6)	381	381	100.00%
Prothrombin time (day -6)	381	381	100.00%
Uric acid (day -6)	381	381	100.00%
Alkaline phosphatase (day -6)	381	381	100.00%
Alanine aminotransferase (day -6)	381	381	100.00%
Aspartate aminotransferase (day -6)	381	381	100.00%
Direct bilirubin (day -6)	381	381	100.00%
Total bilirubin (day -6)	381	381	100.00%
Creatinine (day -6)	381	381	100.00%
Gamma-glutamyl transferase (day -6)	381	381	100.00%
Lactate dehydrogenase (day -6)	381	381	100.00%
Urea (day -6)	381	381	100.00%
Glycated hemoglobin (day -6)	381	381	100.00%
Glucose (day -6)	381	381	100.00%
Albumin (day -6)	381	381	100.00%
Beta-D-glucan (day -6)	381	381	100.00%
C-reactive protein (day -6)	381	381	100.00%
Procalcitonin (day -6)	381	381	100.00%
Total proteins (day -6)	381	381	100.00%
Lactate from arterial blood (day -6)	381	381	100.00%
Lactate from venous blood (day -6)	381	381	100.00%
Triglycerides (day -6)	381	381	100.00%
Basophil cells count (day -7)	381	381	100.00%
Eosinophil cells count (day -7)	381	381	100.00%
Lymphocyte cells count (day -7)	381	381	100.00%
Monocyte cells count (day -7)	381	381	100.00%
Neutrophil cells count (day -7)	381	381	100.00%
Hematocrit (day -7)	381	381	100.00%
Hemoglobin (day -7)	381	381	100.00%
White cells count (day -7)	381	381	100.00%
Red cells count (day -7)	381	381	100.00%
Platelet count (day -7)	381	381	100.00%
Activated partial thromboplastin time (day -7)	381	381	100.00%
International normalized ratio (day -7)	381	381	100.00%
Prothrombin time (day -7)	381	381	100.00%
Uric acid (day -7)	381	381	100.00%
Alkaline phosphatase (day -7)	381	381	100.00%
Alanine aminotransferase (day -7)	381	381	100.00%
Aspartate aminotransferase (day -7)	381	381	100.00%
Direct bilirubin (day -7)	381	381	100.00%
Total bilirubin (day -7)	381	381	100.00%
Creatinine (day -7)	381	381	100.00%
Gamma-glutamyl transferase (day -7)	381	381	100.00%
Lactate dehydrogenase (day -7)	381	381	100.00%

Urea (day -7)	381	381	100.00%
Glycated hemoglobin (day -7)	381	381	100.00%
Glucose (day -7)	381	381	100.00%
Albumin (day -7)	381	381	100.00%
Beta-D-glucan (day -7)	381	381	100.00%
C-reactive protein (day -7)	381	381	100.00%
Procalcitonin (day -7)	381	381	100.00%
Total proteins (day -7)	381	381	100.00%
Lactate from arterial blood (day -7)	381	381	100.00%
Lactate from venous blood (day -7)	381	381	100.00%
Triglycerides (day -7)	381	381	100.00%

* When a test was not performed at a specific time period (e.g., day -1), the automated extraction of a missing value was considered as a correct extraction, since it correctly reflected the lack of information. Pseudonymous information on patient's age, gender, ward of stay, and genus and species of bacteria or *Candida* isolated from all positive blood cultures during the study period were already available in the laboratory information system (LIS). This information can be routinely provided by the laboratory to physicians. Then, the automated extraction system automatically identified the number, origin, and type of episodes among all positive blood cultures, and proceeded with the extraction of all the required variables for the different episodes and organized them in the target dataset.

** Random extractions were performed also to validate the accuracy of the extraction system in distinguishing between contamination and true bacteremia episodes
